# In vitro activity of antimicrobial peptide CDP-B11 alone and in combination with colistin against colistin-resistant and multidrug-resistant *Escherichia coli*

**DOI:** 10.1038/s41598-021-81140-8

**Published:** 2021-01-25

**Authors:** Kaitlin S. Witherell, Jason Price, Ashok D. Bandaranayake, James Olson, Douglas R. Call

**Affiliations:** 1 Allen School, Washington State University, 240 SE Ott Rd., Pullman, WA 99164-7090 USA; 2 Fred Hutchinson Cancer Research Center, Seattle, WA USA

**Keywords:** Microbiology, Antimicrobials, Pathogens

## Abstract

Multidrug-resistant bacteria are a growing global concern, and with increasingly prevalent resistance to last line antibiotics such as colistin, it is imperative that alternative treatment options are identified. Herein we investigated the mechanism of action of a novel antimicrobial peptide (CDP-B11) and its effectiveness against multidrug-resistant bacteria including *Escherichia coli* #0346, which harbors multiple antibiotic-resistance genes, including mobilized colistin resistance gene (*mcr-1*). Bacterial membrane potential and membrane integrity assays, measured by flow cytometry, were used to test membrane disruption. Bacterial growth inhibition assays and time to kill assays measured the effectiveness of CDP-B11 alone and in combination with colistin against *E. coli* #0346 and other bacteria. Hemolysis assays were used to quantify the hemolytic effects of CDP-B11 alone and in combination with colistin. Findings show CDP-B11 disrupts the outer membrane of *E. coli* #0346. CDP-B11 with colistin inhibits the growth of *E. coli* #0346 at ≥ 10× lower colistin concentrations compared to colistin alone in Mueller–Hinton media and M9 media. Growth is significantly inhibited in other clinically relevant strains, such as *Acinetobacter baumannii, Pseudomonas aeruginosa,* and *Klebsiella pneumoniae*. In rich media and minimal media, the drug combination kills bacteria at a lower colistin concentration (1.25 μg/mL) compared to colistin alone (2.5 μg/mL). In minimal media, the combination is bactericidal with killing accelerated by up to 2 h compared to colistin alone. Importantly, no significant red blood hemolysis is evident for CDP-B11 alone or in combination with colistin. The characteristics of CDP-B11 presented here indicate that it can be used as a potential monotherapy or as combination therapy with colistin for the treatment of multidrug-resistant infections, including colistin-resistant infections.

## Introduction

Untreatable strains of multidrug-resistant bacteria have become increasingly common^1^ and it is increasingly difficult to bring new antibiotics to the market in a timely manner^2^. One important antibiotic with declining utilization is colistin, an antibiotic first discovered in the 1940s^3^. Colistin was frequently used in the 1950s to treat Gram-negative infections, but it has been used less frequently in recent decades owing to complications from neuro- and nephrotoxicity and increasing availability of safer alternatives, such as aminoglycosides^3^. With an increasing prevalence of resistance to modern antibiotics, however, colistin is emerging as a last-line antibiotic to treat multidrug-resistant infections^3^.

Colistin acts by binding the outer membrane of Gram-negative bacteria, diffusing into the periplasmic space and further damaging the cytosolic membrane resulting in cell lysis. Unfortunately, plasmid-mediated resistance to colistin has emerged and spread across the globe in just the past few years^4^. The mobilized colistin resistance *(mcr)* gene encodes a phosphoethanolamine transferase that alters 4′ phosphoethanolamine, changing the target of colistin (the lipid A moiety) from a negative to a neutral charge^5^. This prevents colistin from binding efficiently to lipid A and thus prevents the lytic activity of this antibiotic.

Antimicrobial peptides (AMPs) offer a potential means to counteract multiple antibiotic-resistance mechanisms, including *mcr*-mediated resistance to colistin. AMPs typically interact with bacterial membranes to neutralize the membrane charge and physically penetrate the bacterium leading to cell death. This nonspecific mechanism of action allows broad-spectrum activity and is less prone to development of genetically-encoded resistance mechanisms^6^. Nevertheless, some AMPs have a short half-life and the nonspecific nature of their interactions with membranes may include interactions with host cells, which can be indicated by assaying for red blood cell hemolysis^6^. AMPs classified as cystine-dense-peptides are particularly promising drug candidates because they have several intra-disulfide bonds that contribute to their increased stability and more specific binding activity^7^.

Herein we characterized a cystine dense peptide (CDP-B11) to determine whether it can kill or inhibit growth of clinically isolated MDR and colistin-resistant bacteria alone and/or in combination with colistin. We also sought to identify the mechanism of action and ascertain if this peptide causes red blood cell hemolysis. CDP-B11 (UniProtKB accession number A3RJ36) is thought to have bactericidal activity given its similarity to another peptide, Beta defensin 13 (UniProtKB accession number P46171). CDP-B11, is a synthetic version of lingual antimicrobial peptide (LAP), which was originally isolated from a water buffalo. It was previously shown that LAP can be localized to the mammary tissue when there is an infection, such as mastitis, consistent with CDP-B11 being part of the innate immune system^8^. β-defensins are effective against multiple species of bacteria and fungi including *Escherichia coli* K12, *Staphylococcus auerus, Streptococcus pyogenes* and *Candida albicans*^8^. The bovine LAP peptide, isolated from a cow, which differs from CDP-B11 by the addition of an extra N-terminal glutamine and glycine, exhibits antibacterial activity against *E. coli*, *Pseudomonas aeruginosa, S. aureus, C. albicans,* and *Candida tropicalis*^9^. These findings suggest that CDP-B11 can kill or inhibit the growth of bacteria, including colistin- and multidrug-resistant *E. coli,* as well as multidrug-resistant *Acinetobacter baumannii, Pseudomonas aeruginosa, and Klebsiella pneumoniae.*

## Materials and methods

### Bacteria and culture conditions

*E. coli* #0346, *A. baumannii* #0035, *P. aeruginosa* #0054, *E. coli* #0061, and *K. pneumoniae* #0347 are of clinical origin and are archived in the U.S. Food and Drug Administration (FDA)-Center for Disease Control (CDC) Antimicrobial Resistance Isolate Bank^10^. *E. coli* #0346 is multidrug-resistant, harbors several antimicrobial-resistance genes (see Table 1), and is resistant to colistin (minimum inhibitory concentration 4 µg/mL) based on data from the CDC and confirmed by this study (data not shown)*. E. coli* #0346 was cultured in Mueller–Hinton media from Hardy Diagnostics (“rich media”) or in M9 media (Na_2_HPO_4_ 6 g/L, KH_2_PO_4_ 3 g/L, NaCl 0.5 g/L, NH_4_Cl 1 g/L, MgSO_4_ 1 mM, CaCl_2_ 0.1 mM, and 0.2% glucose) supplemented with thiamine (1 mg/L) and leucine (100 μg/mL)^11^ (“minimal media”). *A. baumannii* #0035, *P. aeruginosa* #0054, *E. coli* #0061, and *K. pneumoniae* #0347 harbor multiple antimicrobial resistance genes (see Table 1) and have intermediate resistance to colistin (minimum inhibitory concentration 0.5–1.0 µg/mL)^10^. These strains were cultured in Mueller–Hinton media from Hardy Diagnostics.

**Table 1:** AR isolate bank strains resistance genes and minimum inhibitory concentration (MIC) following CLSI guidelines.

Strain	Antibiotic resistance genes^a^	Colistin MIC (μg/mL)^a^	CDP-B11 MIC^b^ (μg/mL)
*E. coli* AR #0346	*mcr-1, bla*_CMY-2_, *bla*_CTX-M-55_*, aad1_5*, *rmtB*, *strA, strB*, *fosA, mph*(A), *catA1, sul1, sul2, dfrA17*	4	> 200
*A. baumannii* AR #0035	*bla*_TEM-1D,_ *bla*_ADC-25,_ bla_OXA-66,_ *bla*_OXA-72_*, strB, aph(3′)-Ic, strA, mph*(E), *msr*(E), *sul2*	1	25
*P. aeruginosa* AR #0054	*bla*_VIM-4,_ *bla*_OXA-50,_ *bla*_PAO_*, strB, aadB, strA, catB7, tet*(A)	1	> 200
*E. coli* AR #0061	*bla*_KPC-3,_ *bla*_OXA-9,_ *bla*_TEM-1_*, strB, aadA2, aadA1, strA, aac(6′)-Ib, sul1,sul3,sul2, tet*(A)*, dfrA12,dfrA14*	0.5	100
*K. pneumoniae* AR #0347	*aac(6′)-Ib*, *aph(3′)-Ia, dfrA14*, *KPC-3*, *mph*(A)	0.5	> 200

^a^Information from the Center for Disease Control Antimicrobial Resistance Isolate Database. https://wwwn.cdc.gov/arisolatebank/.

^b^Determined by the current study.

### Peptides and antibiotics

Colistin sulfate from Research Product International (lot number 32466–38298) was used for all experiments. Peptide CDP-B11 (VRNSQSCRRNKGICVPIRCPGSMRQIGTCLGAQVKCCRRK) sequence and starting material was produced using previously published methods^7^. Later, the peptide was synthesized as a custom peptide by Biomatik Corporation and demonstrated to match the peptide made by the Fred Hutchinson Cancer research center by high performance liquid chromatography and mass spectrometry. The MSI-78 peptide (H-GIGKFLKKAKKFGKAFVKILKK-OH) was a synthesized by Mimotopes.

### Membrane potential experiments

The Invitrogen BacLight™ bacterial membrane potential kit (Invitrogen, Thermo Fisher Scientific) uses DiOC_2_ as a fluorescent reporter and 500 μM carbonyl cyanide m-chlorophenyl hydrazine (CCCP) as a positive control^12^. DiOC_2_ exhibits green fluorescence when cells have limited membrane potential and red fluorescence in the presence of high membrane potential. Briefly, subcultures were made from overnight cultures and grown to the beginning of log phase. Cells were then incubated for 1 h, without shaking at 37 °C, with either no treatment, peptide alone, colistin alone, or a combination of peptide and colistin. Cells were then diluted 10^–2^ with PBS (to ~ 1 × 10^6^ cells/mL). CCCP was added as a positive control to the no-treatment culture. DiOC_2_ (10 μL of 3 mM stock solution) was added to each sample (except the unstained controls). Samples were incubated for 5–15 min and analyzed on a FACSCalibur™ (BD Biosciences). Forward scatter (FSC), side scatter (SSC), FL1 (488/530), and FL3 (488/613) were used to detect fluorescent dyes.

Analysis was completed by using Kaluza, Flow Cytometry Analysis Software by Beckman Coulter. Cells were gated on high FSC and high SSC. Gated cells were graphed on FL1 vs. FL3. Cells were gated on red and green cell populations and values were calculated as (number of red cells)/(number of green cells). A one-way ANOVA was used to compare all samples to the no-treatment control.

### Membrane integrity experiments

The TO-PRO-3 Ready Flow reagent (Invitrogen, Thermo Fisher Scientific) was used to assess membrane integrity. TO-PRO-3 Ready Flow is a fluorescent dye that stains double-stranded DNA, but it can only do so after penetrating a damaged bacterial membrane. Briefly, samples were prepared as described for membrane potential experiments. Two drops of TO-PRO-3 Ready Flow reagent were added to each sample and samples were incubated for 15 min before analysis with FACSCalibur™ system. CCCP was used as a negative control and 1.25 μg/mL MSI-78 was used as a positive control. Cells were gated on high FSC vs. high SSC. Cells were then gated above 10^–2^ on a log scale. Treatments were compared with the negative control by using a one-way ANOVA correcting for multiple comparisons using a post hoc Dunnett’s correction.

### Growth inhibition experiments

Growth inhibition assays were used to generate growth curves of *E. coli #0346* over a 22-h period. Growth inhibition was defined as the concentration of compound that inhibited growth as measured by an OD_600_, to be equivalent to the sterility control. The results of the growth inhibition assay for each organism were determined by making twofold serial dilutions of each compound at varying concentrations (in Mueller–Hinton rich media or M9 minimal media plus glucose). Bacterial cultures were diluted 1:10,000 from ~ 0.8 OD_600_. The diluted cultures were then added with antibiotic to wells of 100-well honeycomb plates. The plates were incubated in a Labsystems Bioscreen C plate reader for 22 h, taking the OD_600_ measurement every hour at 37 °C (shaking continuously). From the growth curve data, a growth inhibition concentration (GIC) was estimated. The GIC was the estimated concentration at which the OD_600_ endpoint value for the treatment was significantly inhibited compared to the nontreated growth control as determined by a Student’s *t*-test with the Holm–Sidak method, with α = 0.05. This method of comparison allows for analysis of the growth curve over time to observe changes in log-phase and final optical density. This assay differs from the MIC assays because it documents changes to rate of growth over time rather than just the concentration of compound that completely inhibits population growth. Every experiment included no-treatment bacterial culture and media-only controls for sterility and background measurements. At least three independent experiments were completed with ≥ 2 technical replicates for each antibiotic treatment. Significance was determined by comparing the OD_600_ of CDP-B11 plus colistin to the OD_600_ of colistin alone for each time point using multiple Student *t*-tests. Compounding error was controlled by using the Holm–Sidak method, with α = 0.05. Each row was analyzed individually, without assuming a consistent standard deviation.

### Combination experiments

Combinatorial assays were performed with a fixed concentration of CDP-B11 and twofold serial dilutions of colistin. A checkerboard assay was used to select the GIC that reflected the greatest combinatorial effect, while not showing significant growth inhibition between peptide alone and the growth control after 20 h incubation. Controls included CDP-B11 only, bacterial culture only (growth control), and Mueller–Hinton media or M9 media only (sterility/background control). The plates were incubated in a Labsystems Bioscreen C plate reader for 20 (*E. coli* #0346) or 24 (*A. baumannii* #0035, *P. aeruginosa* #0054, *E. coli* #0061, and *K. pneumoniae* #0347) hours, collecting the OD_600_ value every hour at 37 °C and shaking continuously.

Statistical analysis of combinatorial assays was performed using Graphpad PRISM 8.2.1. A significant fold reduction for drug combination experiments was determined by normalizing OD_600_ values at 0 and 20 or 24 h (to remove background absorbance) and the colistin concentration for which growth culture turbidity (OD_600_) was > 0.05 was recorded. Each measurement was replicated independently ≥ 3 times, with ≥ 2 technical replicates. Statistical comparisons were performed with the corresponding Holm-Sidak method and α ≤ 0.05, without assuming equal variance. Significant fold reduction was tallied using the formula 2^n^, where n is the number of twofold colistin dilutions tested with significant differences for CDP-B11 plus colistin compared to the colistin alone. Fold reduction was considered biologically significant if 2^n^ ≥ 4.

### Minimum inhibitory concentration (MIC) broth microdilution assays

Conventional MIC assays followed the guidelines described by the Clinical Laboratory and Standards Institute (CLSI)^13^. Briefly, overnight cultures were adjusted to an optical density of 0.8 (600 nm) and then diluted (10–4) in Mueller–Hinton media and 50 μL of diluted culture were added to each well in Greiner 96-well, U-bottom microplates (Greiner, MilliporeSigma). Dilution series for final concentrations ranging between 0 and 8 μg/mL for colistin between 0 and 200 μg/mL for CDP-B11. The final volume in each well was 100 μL. The plates were incubated at 37 °C (stationary) for ~ 18 h. Aliquots (5 μL) were taken from each well and plated onto LB agar plates and grown at 37 °C overnight. Plates were examined for growth and the lowest treatment concentration with no growth was considered the MIC. At least two independent assays were completed and each assay included two technical replicates.

### Time to kill assays

Bacterial cultures were set up as described for combination experiments. For Mueller–Hinton media, 5 μL aliquots of culture (in triplicate) were taken every hour from 0 to 24 h. For M9 media, 5 μL aliquots of culture (in triplicate) were taken every hour from 0 to 8 h, 12, and 24 h, and plated on LB agar. Plates were incubated overnight at 37 °C and colonies counted in the morning. If colonies were uncountable (≥ 100 colonies/5 μL), then the aliquot was labeled “Growth”. The time to kill (or Lethal Dose_99_) was defined as the hour at which an average of < 1 colony was recovered.

### Hemolysis experiments

The hemolysis assay was adapted from Monteiro et al.^14^. Defibrinated sheep blood (100 mL; < 2 weeks old; Hardy Diagnostics) was centrifuged (500×*g* at 4 °C, 15 min) and resuspended in 10-mL PBS. This process was repeated three times before red blood cells (RBCs) were enumerated with a hemocytometer and diluted to 1 × 10^9^ RBC/mL in PBS. Diluted RBC (100 μL) was aliquoted into each well of a 96-well white polystyrene plate (Thermo Fisher Scientific). The plate was centrifuged at 500×*g* at 4 °C (10 min) and the supernatant aspirated from every well before adding 100 μL of a treatment to the RBC pellet. PBS (100 μL) was used as a negative control and 0.1% TritonX-100 (100 μL) was used as a positive control. Cells were incubated at 37 °C for 1 h and then centrifuged at 500×*g* at 4 °C (10 min). Supernatant was then transferred into a flat bottom clear 96-well plate and the OD_450_ was recorded with a Tecan Spark plate reader. Readings were normalized using the equation (treatment-negative control)/(positive control-negative control)*100 to calculate the percent hemolysis in each well. Three independent experiments were performed with duplicates.

## Results

### CDP-B11 reduces membrane potential and membrane integrity

**Figure 1:**
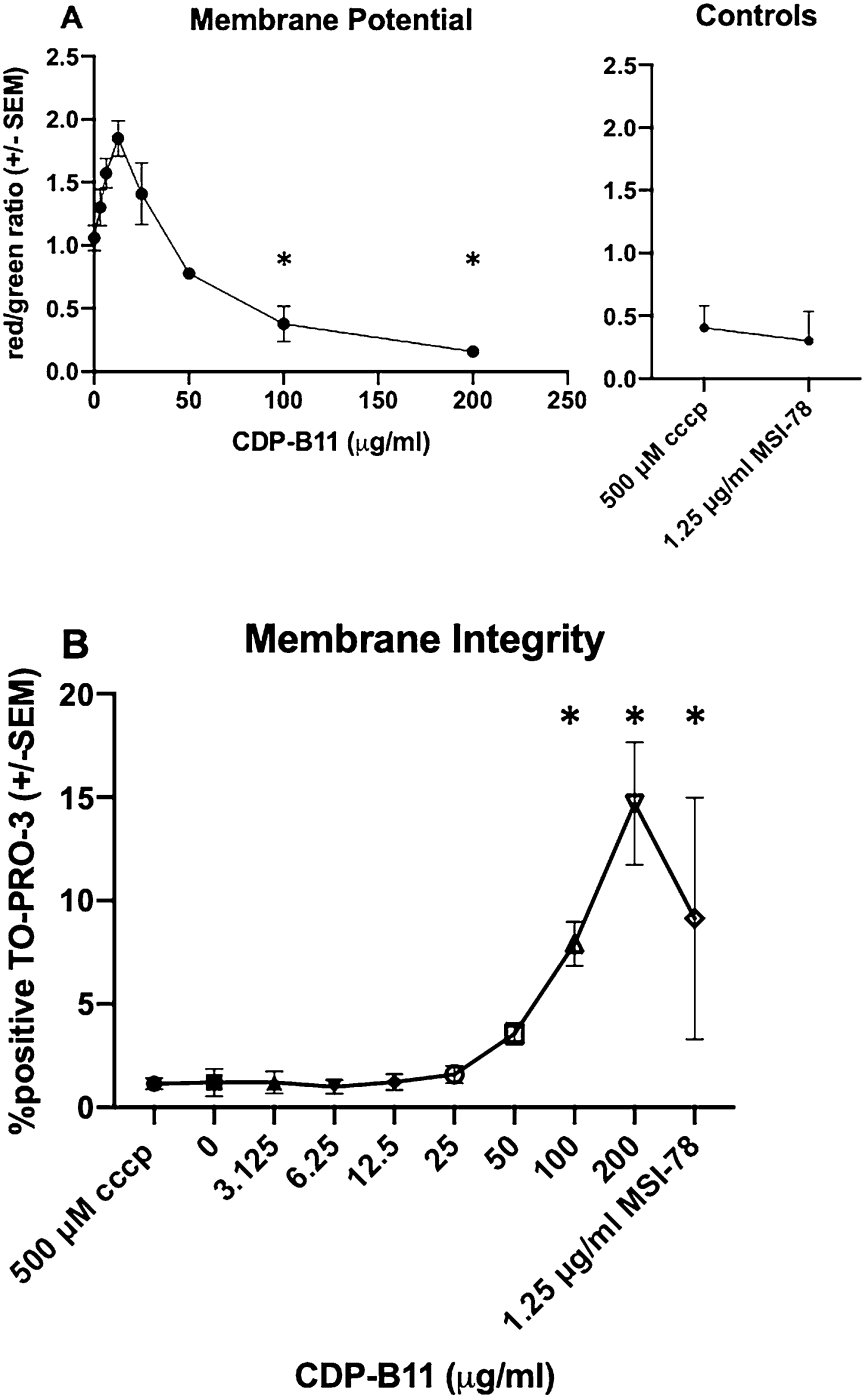
Assessment of membrane integrity. (**A**) Red/green ratio of cells relative to differing concentrations of CDP-B11. Both CCCP (500 µM) and peptide MSI-78 (1.25 μg/mL) were used as positive controls. (**B**) Fluorescent signal of cells treated with peptide CDP-B11. Higher signal corresponds to greater membrane damage. CCCP was used as a negative control because it reduces membrane potential without causing membrane damage. MSI-78 (1.25 μg/mL) served as a positive control for membrane damage. Results were analyzed using one-way ANOVA correcting for multiple comparisons using a post hoc Dunnett’s correction for multiple comparisons (*P* < 0.05).

Initial experiments indicated that *E. coli* #0346 was susceptible to CDP-B11 so we used this as the model strain to determine if CDP-B11 disrupts bacterial membrane potential. This was assessed by using a commercial kit (Baclight bacterial membrane potential kit) for which a lower red/green ratio indicates reduced membrane potential. Because this assay requires samples from a robust log phase of growth, only Mueller–Hinton media was used. With lower concentrations of CDP-B11 (< 50 μg/mL), membrane potential increased but this was reversed with higher concentrations (Fig. 1A). The ability of CDP-B11 to disrupt bacterial membrane integrity was assessed with a fluorescent dye (TO-PRO-3 Ready Flow) whereby increasing fluorescent signal indicates decreasing membrane integrity. Fluorescent signal increased with the addition of > 25 μg/mL CDP-B11 (Fig. 1B).

**Table 2:** Time kill assay results for *E. coli* #0346 in Mueller–Hinton and M9 media.

Conc. colistin (μg/mL)	Hour LD_99_			
	Mueller–Hinton		M9	
	50 μg/mL CDP-B11 plus colistin	Colistin only	50 μg/mL CDP-B11 plus colistin	Colistin only
0	Growth	Growth	Growth	Growth
1.25	14	Growth	6	Growth
2.5	3	3	4	6
5	2	2	3	5
10	1	1	3	3

### CDP-B11 inhibits bacterial growth

**Figure 2:**
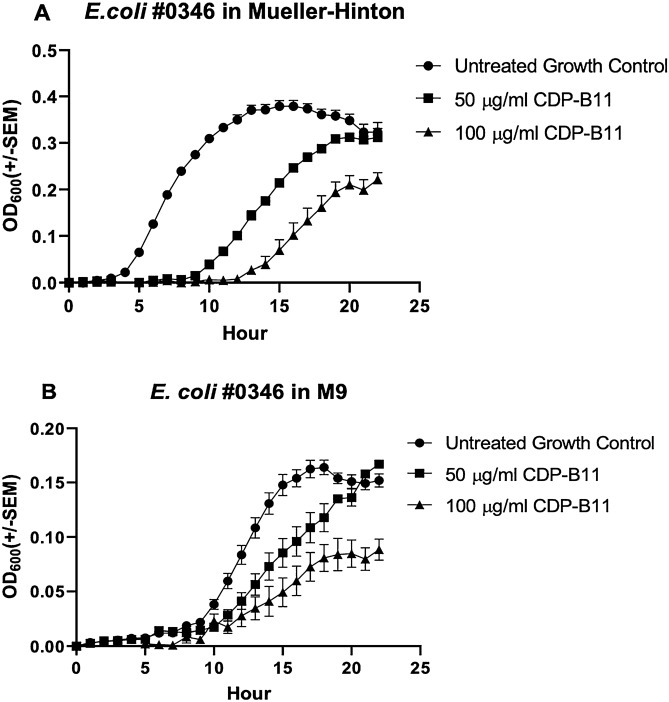
Growth curves (OD_600_) for *E. coli* #0346 over 22 h. No treatment (circles), with CDP-B11 (50 μg/mL, squares; 100 μg/mL, triangles) using (**A**) Mueller–Hinton media, or (**B**) M9 media.

CDP-B11 (50 and 100 μg/mL) delays and inhibits growth of *E. coli* #0346 in both Mueller–Hinton and M9 media. In Mueller–Hinton media, the difference in growth was significant for 50 μg/mL CPD-B11 (4–19 h) and 100 μg/mL CDP-B11 (4–20 h) (Fig. 1A). In M9 media, the difference in growth was significant for 50 μg/mL CPD-B11 (10–18 h) and 100 μg/mL CDP-B11 (1–2 h, 9 h, and 11–22 h (Fig. 2B). CDP-B11 (50 and 100 μg/mL) affected the log phase of bacterial growth in both minimal and rich media (Fig. 2A,B), but only affected the end of the stationary growth phase at 100 μg/mL. Importantly, bacteria were recovered on LB agar plates for the no-treatment control, 50 μg/mL CDP-B11, and 100 μg/mL CDP-B11 treatment groups indicating that at these concentrations, CDP-B11 is bacteriostatic at these concentrations.

### Combining subinhibitory concentrations of colistin with a subinhibitory concentration of CDP-B11 results in increased efficacy of both colistin and CDP-B11

**Figure 3:**
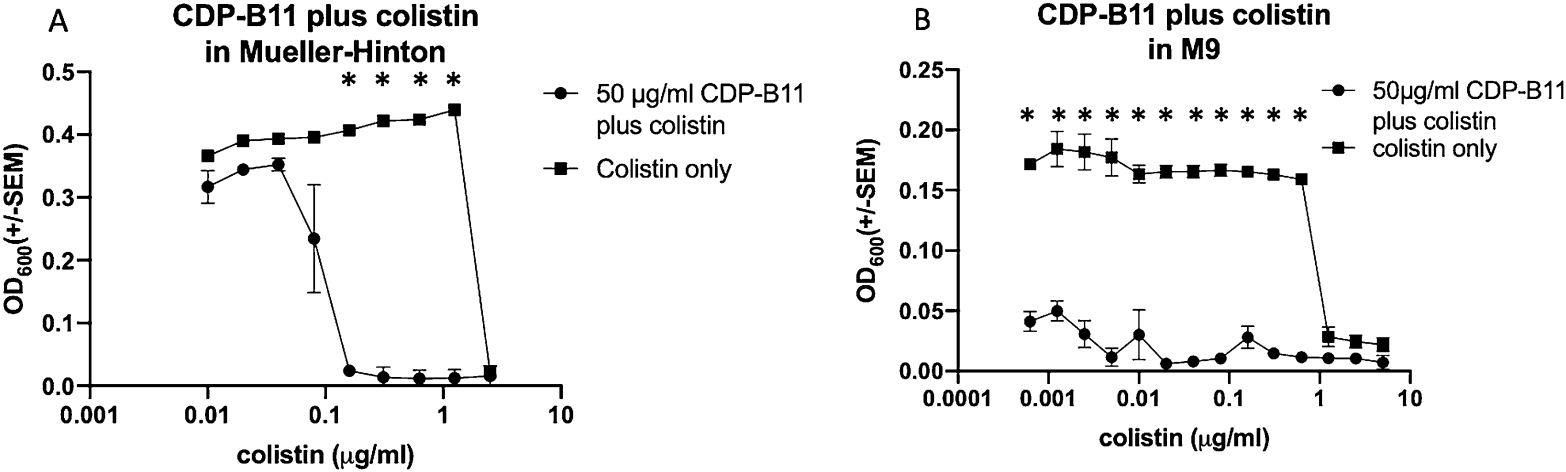
Optical density (600 nm) after 20 h culture for *E. coli* AR #0346 with both CDP-B11 and colistin in (**A**) Mueller–Hinton media, or (**B**) M9 media. Circles represent colistin concentration with a fixed concentration (50 μg/mL) of CDP-B11. Squares represent cultures treated with colistin only. Statistical comparisons were performed with the corresponding Holm–Sidak method and α ≤ 0.05, without assuming equal variance.

For combinatorial experiments, a fixed concentration of CDP-B11 at 50 μg/mL was used. A concentration of 50 μg/mL was selected because the endpoint OD_600_ measurement was not significantly different from the growth control. This allows the combinatorial effect to be evaluated as a statistically significant difference in endpoint values. In Mueller–Hinton media, combining CDP-B11 (at a fixed dose of 50 μg/mL) with colistin reduced the minimum inhibitory concentration of colistin from 2.5 to 0.156 μg/mL (Fig. 3A), equivalent to a 2^4^ or 16-fold reduction. In M9 media, CDP-B11 with colistin reduced the MIC of colistin from 1.25 to 0.0005 μg/mL, equivalent to a 2^11^ or 2,048-fold reduction (Fig. 3B). Importantly, when cultures were plated on LB plates after the growth experiment, no growth was observed at any of the statistically significant concentrations, indicating that CDP-B11 plus colistin is bactericidal (data not shown).

### The CDP-B11 plus colistin combination affects multiple strains

**Figure 4:**
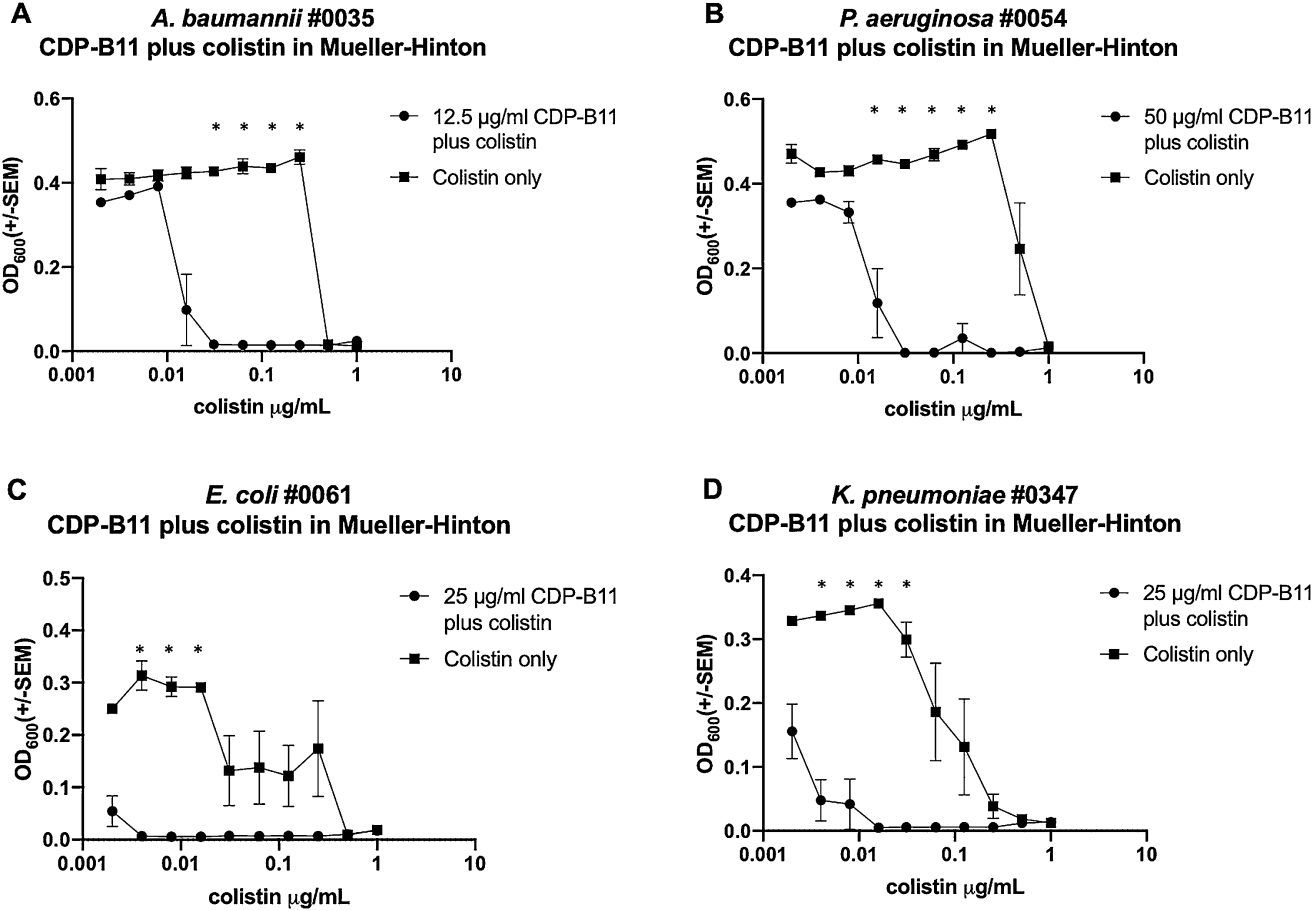
Optical density (600 nm) after 24 h culture for *A. baumannii* #0035 (**A**), *P. aeruginosa* #0054 (**B**), *E. coli* #0061 (**C**), and *K. pneumoniae* #0347 (**D**) with both CDP-B11 and colistin in Mueller–Hinton media. Circles represent colistin concentration with a fixed concentration of CDP-B11. Squares represent cultures treated with colistin only. Statistical comparisons were performed with the corresponding Holm–Sidak method and α ≤ 0.05, without assuming a consistent standard deviation.

CDP-B11 plus colistin combinatorial experiments were performed using *A. baumannii* #0035, *P. aeruginosa* #0054, *E. coli* #0061, and *K. pneumoniae* #0347 to determine the efficacy of the CDP-B11 plus colistin combination against multiple colistin intermediately susceptible species. *A. baumannii* #0035 showed a 2^4^ or 16-fold reduction when 12.5 μg/mL of CDP-B11 was added (Fig. 4A). *P. aeruginosa* #0054 showed a 2^5^ or 32-fold reduction when 50 μg/mL of CDP-B11 was added (Fig. 4B). *E. coli* #0061 showed a 2^3^ or eightfold reduction when 25 μg/mL of CDP-B11 was added (Fig. 4C). *K. pneumoniae* #0347 showed a 2^4^ or 16-fold reduction when 25 μg/mL of CDP-B11 was added (Fig. 4D).

### Minimum inhibitory concentrations found for CDP-B11

*A. baumannii* #0035 and *E. coli* #0061 exhibited an MIC of 25 μg/mL and 100 μg/mL respectively. No MIC was found for CDP-B11 up to 200 μg/mL for *E. coli* #0346, *P. aeruginosa* #0054, and *K. pneumoniae* #0347 (Table 1).

### Combining CDP-B11 and colistin can reduce time-to-kill and lethal concentrations

In Mueller–Hinton media, 50 μg/mL CDP-B11 plus colistin killed *E. coli* #0346 at lower concentrations than colistin alone, but not at a faster rate as shown by CFU. At 1.25 μg/mL colistin, CDP-B11 plus colistin killed > 99% of bacteria (LD_99_) by hour 14 at a colistin concentration of while colistin alone had no activity at this dose. At colistin concentrations of 2.5, 5, and 10 μg/mL, the combination and colistin alone reached LD_99_ at the same rate (Table 2). In M9 media, 50 μg/mL CDP-B11 plus 1.25 μg/mL colistin reached LD_99_ by hour 6, while colistin alone had no activity at this dose. The combination reached LD_99_ faster at colistin concentrations 2.5 and 5 μg/mL. At colistin concentration of 10 μg/mL, CDP-B11 plus colistin and colistin alone both killed bacteria at 3 h (Table 2). At 50 μg/mL CDP-B11 alone and 1.25 μg/mL colistin alone, there was growth at every time point from 0 to 24 h (CFU data available in Supplementary Table 1). For rich media, the difference between CDP-B11 plus colistin and colistin alone was only evident at 1.25 μg/mL, where the combination was able to kill the bacteria and colistin alone was not. The bacterial killing effect was more pronounced in minimal media whereby the time to kill was shorter when CDP-B11 was added to colistin except at 10 μg/mL. Overall, a combinatorial bacterial killing effect was more pronounced in minimal media compared to rich media.

### CDP-B11 plus colistin does not increase red blood cell hemolysis

**Figure 5:**
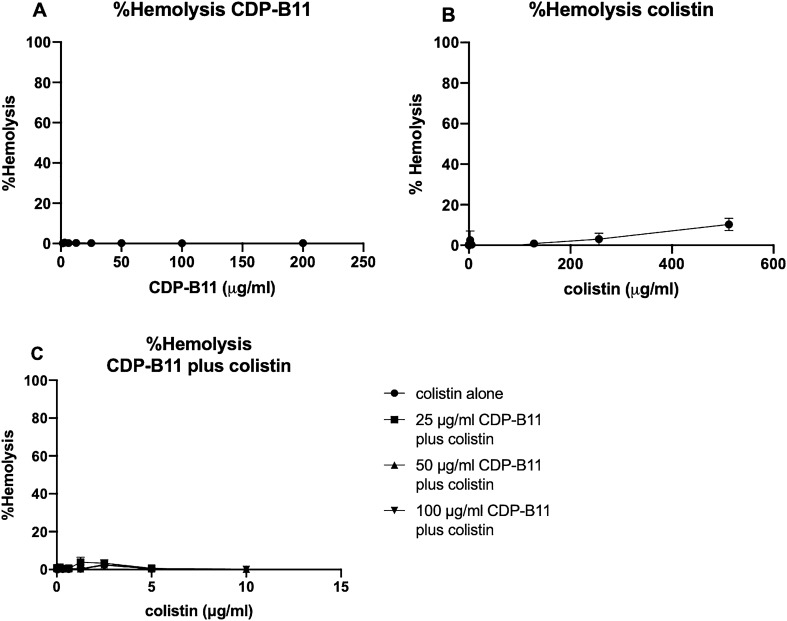
Percent red blood cell hemolysis of CDP-B11 alone, colistin alone or CDP-B11 plus colistin. (**A**) Percent red blood cell lysis of CDP-B11 alone; (**B**) Percent red blood cell hemolysis of colistin alone; (**C**) Percent red blood cell hemolysis of CDP-B11 plus colistin.

Red blood cell (RBC) hemolysis is traditionally used as a preclinical indicator of toxicity. Colistin alone hemolyzed red blood cells at concentrations > 100 μg/mL (Fig. 5A), while no significant RBC hemolysis was observed for CDP-B11 alone (up to 200 μg/mL, or 4.47^−5^ M) (Fig. 5B). When colistin (10–0.01 μg/mL) was combined with CDP-B11 (100–25 μg/mL), no significant RBC hemolysis was observed in a checkerboard assay (Fig. 5C). The percentage of RBC hemolysis did not increase when CDP-B11 and colistin were combined compared to the RBC percent hemolysis of CDP-B11 and colistin alone.

## Discussion

In this study, we were able to demonstrate that the antimicrobial peptide CDP-B11 inhibits bacteria by depolarizing and damaging bacterial membranes. When combined at subinhibitory concentrations, the efficacy of both CDP-B11 and colistin were enhanced significantly. Furthermore, the CDP-B11 plus colistin combination did not increase hemolysis compared to using either CDP-B11 or colistin alone at the tested concentrations.

Given that many AMPs kill or inhibit growth of bacteria by interacting with the bacterial membrane, we tested whether membrane potential and membrane integrity were affected by CDP-B11^15^. At low concentrations of CDP-B11, membrane potential appeared to increase, but we surmise that this was an experimental artifact resulting from the initial addition of the positively-charged peptide into the solution. Because bacterial membrane potential is relative between a cell and its environment, the addition of low concentrations of B11 may cause a slight increase in relative membrane potential at concentrations lower than what is sufficient to actually disrupt the membrane. At high CDP-B11 concentrations (100 and 200 μg/mL), bacterial membrane potential decreased significantly, and membrane permeability increased significantly, indicating that CDP-B11 depolarizes the bacterial membrane to create pores, causing membrane destabilization and loss of membrane integrity.

Given that CDP-B11 appears to destabilizing the bacterial membrane, we investigated if combining CDP-B11 with another membrane-damaging compound would result in a synergistic effect. To examine synergy, a fixed concentration of CDP-B11 (50 μg/mL), that produced no significant growth inhibition in *E. coli* #0346, was combined with serially diluted colistin. Bacterial growth for CDP-B11 plus colistin was compared to colistin alone under robust (Mueller–Hinton rich media) and stressful (M9 minimal media) conditions. In both media types, the combination of CDP-B11 and colistin appeared synergistic. However, synergy was much more evident under stress conditions compared to more robust growth conditions, possibly because bacteria must upregulate biosynthetic pathways in order to survive on a single carbon source in minimal media^16^. The latter can cause the bacteria to grow at a much slower rate, potentially making them more sensitive to antibiotic treatment.

To explore the synergistic nature of CDP-B11 plus colistin in other bacteria, we used the combinatorial experiment with four clinically relevant, colistin intermediately susceptible strains of *A. baumannii, P. aeruginosa, E. coli, and K. pneumoniae.* In the presence of both CDP-B11 and colistin, all four strains exhibited an 8- to 32-fold increase in susceptibility when compared to colistin alone. These findings indicate that the CDP-B11 plus colistin is effective against multiple clinically relevant species in vitro. These findings also indicate that the CDP-B11 plus colistin combination is effective against strains that have a wide range of colistin resistance.

In Mueller–Hinton rich media*,* CDP-B11 plus colistin killed *E.* *coli* #0346 at lower colistin concentrations, but at the same rate compared to colistin alone. In M9 minimal media, CDP-B11 and colistin exhibited synergy in both growth assays and time kill assays. CDP-B11 plus colistin killed *E.* *coli* #0346 at lower colistin concentrations and at a faster rate compared to colistin alone. Being able to kill bacteria or inhibit bacterial growth at lower concentrations by using combination therapy may allow for faster recovery rate and reduced toxic side effects.

When CDP-B11 alone is used at high concentrations (≥ 100 μg/mL) in *E. coli* #0346, growth is significantly inhibited. It is important to note in the bacterial growth curve, the log phase of bacterial growth is delayed and significantly flattened. This is an important finding because reduced bacterial growth rate may allow the patient’s immune system to successfully fight the infection.

For *E. coli* #0346, the reported MIC for colistin (4 μg/mL) was confirmed in Mueller–Hinton. However, the time kill assay showed colistin in Mueller–Hinton media at 2.5 μg/mL which is lower, but not significantly lower (> 1 dilution factor), than the MIC value. The different values between the colistin MIC assay and time kill assay could be attributed to the different types of plastic used in the Greiner plates (for MIC) versus the Bioscreen C plates (for time kill assays and growth inhibition assays), as colistin can interact differently with different types of plastic^17,18^. This difference may also be due to a difference in the bioavailability of colistin in the assays. The Bioscreen C plates were shaken for the growth inhibition assays, but not for the time kill assays. The Greiner plates used in the MIC assays were not shaken. The shaking might allow colistin to bind to more bacterial cells before binding to the plastic of the plate, resulting in a lower effective concentration of colistin. This change could also be an experimental artifact given the difference is not significant.

Unlike some other AMPs, such as Pexiganan (MSI-78)^19^, CDP-B11 does not cause hemolysis of red blood cells. A hemolysis assay was used as a preliminary method to evaluate the potential toxicity of CDP-B11^14^. No hemolysis was evident for CDP-B11 and no increase in hemolysis was observed when CDP-B11 was combined with colistin. Several AMPs have been tested in in vivo studies and show promise as viable antibacterial agents. Peptide DGL13K was shown to treat burns infected with *Pseudomonas aeruginosa* in mice without toxic effects^20^. Similarly, “Peptide 11”, a non-natural peptide, described by Boullet et al. showed approximately 50% survival rate against septic in mice compared to a 0% survival rate in the control population^21^. These peptides show proof of concept that AMPs, either natural or non-natural can work in vivo. The major limitation to using AMPs as alternative therapeutics is not efficacy, but rather the cost of scaling up production.

This study suggests that CDP-B11 may be an attractive alternative to traditional antibiotics against colistin-resistant and multidrug-resistant bacteria. As shown by the Baclight membrane potential kit and the TO-PRO-3 ready flow reagent, CDP-B11 functions by decreasing membrane potential and destroying membrane integrity. Because of this mechanism of action, CDP-B11 may be a good candidate for combination therapy with other antibacterials that damage the membrane, such as colistin. Because colistin monotherapy has harmful side effects including neurotoxicity and nephrotoxicity^22^, a combinatorial approach may reduce the dose of colistin necessary to treat a patient thereby reducing the toxicity of colistin while enhancing its efficacy^23^. Consequently, the combination of colistin and CDP-B11 may improve the safety profile of colistin with lower clinically-relevant concentrations of colistin and/or shorter treatment regimens, and could expand potential applications of colistin therapy.

## Supplementary Information

Supplementary Information.

## Data Availability

All data not already present in the manuscript can be made available upon request.
