# Supplementary material for: In vitro activity of antimicrobial peptide CDP-B11 alone and in combination with colistin against colistin-resistant and multidrug-resistant Escherichia coli
Source: Sci Rep. 2021 Jan 25;11:2151. doi: 10.1038/s41598-021-81140-8 (PMC7835343; doi:10.1038/s41598-021-81140-8)
Supplement: Supplementary file 1 — Supplementary Information. [file 41598_2021_81140_MOESM1_ESM.pdf]

Supplemental Table 1

In vitro activity of antimicrobial peptide CDP-B11 alone and in combination with colistin against colistin-resistant and multidrug-resistant *Escherichia coli*

Kaitlin S. Witherell

Jason Price

Ashok D. Bandaranayake

James Olson

Douglas R. Call

Supplementary Table 1A: Average colony forming units (CFU) present at each hour for colistin only or 50 µg/ml CDP-B11 plus colistin in Mueller-Hinton.

	Colistin only					50 µg/ml CDP-B11 plus colistin				
µg/ml	0	1.25	2.5	5	10	0	1.25	2.5	5	10
0	>100	>100	>100	>100	>100	>100	>100	>100	>100	>100
1	>100	>100	>100	0	0	>100	>100	>100	<1	0
2	>100	>100	5.3	0	0	>100	>100	2.3	0	0
3	>100	>100	0	0	0	>100	>100	0	0	0
4	>100	>100	<1	0	0	>100	>100	<1	0	0
5	>100	>100	0	0	0	>100	>100	0	0	0
6	>100	>100	0	0	0	>100	>100	0	0	0
7	>100	>100	0	0	0	>100	>100	0	0	0
8	>100	>100	0	0	0	>100	>100	0	0	0
9	>100	>100	0	0	0	>100	>100	0	0	0
10	>100	>100	0	0	0	>100	>100	0	0	0
11	>100	>100	0	0	0	>100	>100	0	0	0
12	>100	>100	0	0	0	>100	>100	0	0	0
13	>100	>100	0	0	0	>100	13, 11	0	0	0
14	>100	>100	0	0	0	>100	0	0	0	0
15	>100	>100	0	0	0	>100	0	0	0	0
16	>100	>100	0	0	0	>100	0	0	0	0
17	>100	>100	0	0	0	>100	0	0	0	0
18	>100	>100	0	0	0	>100	0	0	0	0
19	>100	>100	0	0	0	>100	0	0	0	0
20	>100	>100	0	0	0	>100	0	0	0	0
21	>100	>100	0	0	0	>100	0	0	0	0
22	>100	>100	0	0	0	>100	<1	0	0	0
23	>100	>100	0	0	0	>100	0	0	0	0
24	>100	>100	0	0	0	>101	0	0	0	0

Supplementary Table 1B: Average colony forming units (CFU) present at each hour for colistin only or 50 µg/ml CDP-B11 plus colistin in M9+Glucose media.

	Colistin only					50 µg/ml CDP-B11 plus colistin				
µg/ml	0	1.25	2.5	5	10	0	1.25	2.5	5	10
0	>100	>100	>100	>100	>100	>100	>100	>100	>100	>100
1	>100	>100	>100	>100	3.3	>100	>100	>100	>100	5.3
2	>100	>100	5.0	4.3	1.6	>100	>100	6.9	3.4	2
3	>100	>100	<1	0.0	0.0	>100	11	2.1	<1	<1
4	>100	>100	<1	1.3	0	>100	1.6	<1	<1	0
5	>100	>100	3.8	<1	0.0	>100	2.2	1	<1	<1
6	>100	>100	<1	<1	<1	>100	<1	<1	<1	<1
7	>100	>100	1.0	<1	0	>100	<1	<1	<1	0
8	>100	>100	<1	<1	0	>100	<1	<1	0	0
12	>100	>100	0	0	0	>100	0	0	0	0
24	>100	>100	0	0	0	>100	0	0	0	0
